# A Novel approach to catheterizing the Left Gastric Artery for bariatric embolization

**DOI:** 10.1016/j.radcr.2021.08.051

**Published:** 2021-09-16

**Authors:** Mubin Syed, Wajahat Efridi, Tanya Khan, Feras Deek, Matthew Kurian, Azim Shaikh

**Affiliations:** a Dayton Interventional Radiology, 3075 Governors Place Blvd., Ste. 120, Dayton, OH 45409, USA; b Samaritan Hospital, 2222 Philadelphia Dr., Dayton, OH 45406, USA; c Boonshoft School of Medicine at Wright State, 3640 Colonel Glenn Hwy, Dayton, OH 45435, USA; d Springfield Regional Medical Center, 100 Medical Center Drive, Springfield, OH 45504, USA; e Northeastern Ohio Medical University (NEOMED), 4209 OH-44, Rootstown, OH 44272,USA; f Suny Upstate Medical University, 750 E Adams Street, Syracuse, NY, 13210

## Abstract

We present the case of a 45 Y/o morbidly obese male who - unable to lose weight despite extensive lifestyle interventions, presented to us for an experimental procedure known as bariatric embolization. This arterial embolization procedure was performed targeting the left gastric artery with aims to restrict blood flow to the gastric fundus, and lowering serum ghrelin levels. In this case the left gastric artery arose cephalad from the celiac, earlier than where it normally branches; a variation known as a “false tripod”, causing some initial difficulty with selection. We describe how introducing a tertiary curve on the guidewire proved successful. Adapting the guidewire for difficult anatomy is an often a very underutilized skillset for many interventionalists - but led to success in this rather nascent procedure.

## Introduction

Since the 1970s, embolization of the Left Gastric Artery has been an established technique to treat gastric bleeding. [1,2] In fact, it was so safe that it could even be performed empirically without angiographic evidence of extravasation. [3] Given the stomach's extensive collateral blood supply, necrosis is rare but “back-door” bleeding can occur.[4]

Retrospectively, significant weight loss was noted to be an inadvertent side effect in this procedure. [5] This finding paved way for left gastric artery embolization (bariatric embolization) for obesity.

While the exact mechanism of weight loss is unknown, the effect is attributed to some ischemic insult sustained to the fundus of the stomach. Most patients following this procedure experience a drop-in serum ghrelin; also known as the “hunger hormone”, a powerful mediator of hunger and satiety.

Selective catheterization of the left gastric artery can be difficult, and we present a wiring solution for a common anatomical variation, the “false tripod” with posterior angulation of the left gastric artery.

## Case report

A 45 year old Male with no significant past medical history other than morbid obesity presented to us for an experimental procedure known as bariatric embolization. At 6’2” and 277 lbs. (BMI of 35.6); he failed attempts for weight loss with conservative management including diet, exercise, and behavior modification, thus deemed a prime candidate for enrollment into our program.

We scheduled him to undergo a left gastric artery embolization for bariatric weight loss and appetite suppression. He was started on prophylactic dosing of Omeprazole (40mg bid) and Sucralfate a week and a day prior respectively.

Opting for moderate sedation with midazolam and propofol, we gave him 1L of LR, Ondansetron, and Pantoprazole as pre-medication and obtained femoral access with a 21-gauge needle under sonography and used a 5-Frech VCF catheter for an Abdominal Aortic angiogram, showing us wide patency in the proximal celiac artery.

A 5-French Mickelson catheter was used for celiac angiography. Here the splenic, common hepatic, proper hepatic, and most importantly the left gastric arteries were all identified and appeared widely patent. Here we noted on how the left gastric artery emanated superior turning posteriorly with respect to the celiac trunk, and tried pushing a .014 in. PROWATER curved-tip guidewire in with limited success.

We switched to a coaxial PROGEAT microcatheter (Terumo Medical, Japan) in various bilateral anterior oblique projections, [Fig. 3] and established a secondary curve on the guidewire for entry - all without avail. We brought out an angled CXI support catheter (Cook Medical, Bloomington, IN) [Fig. 4] again without success. Finally, after placing a tertiary curve on the wire [Fig. 5], on lateral projection, we got entry into the left gastric artery, totaling just under 30 minutes of fluoroscopy time.

**Fig. 1:**
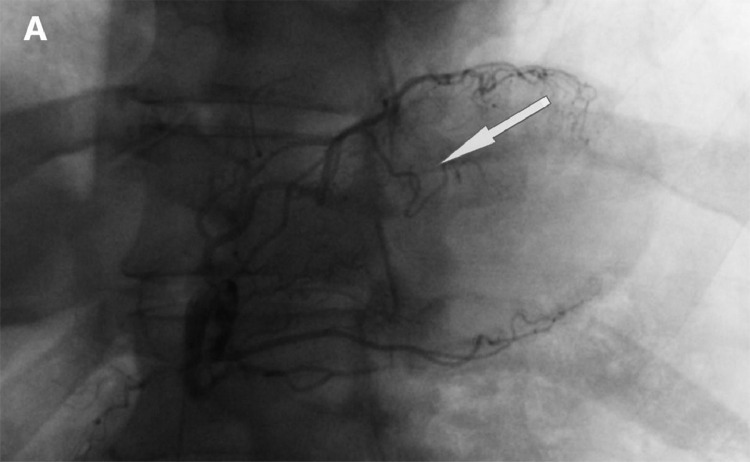
Left gastric angiogram PRE-embolization, the superior branch of the left gastric artery (Black arrow) perfuses almost completely into the fundus and lower esophageal sphincter

A DIREXION HI-FLO microcatheter was advanced into the Left Gastric and hand injection in the AP view confirmed its patency. A cocktail of nitroglycerin, Heparin, and 2.5mg Verapamil were instilled before installing one complete vial of 300-500um beads for stasis. There was mild anterograde flow [Fig. 1+2] but the contrast washed away after 7-8 cardiac pulsations, achieving stasis. The patient was sent home after hydration, with follow-up at 6mo.

**Fig. 2:**
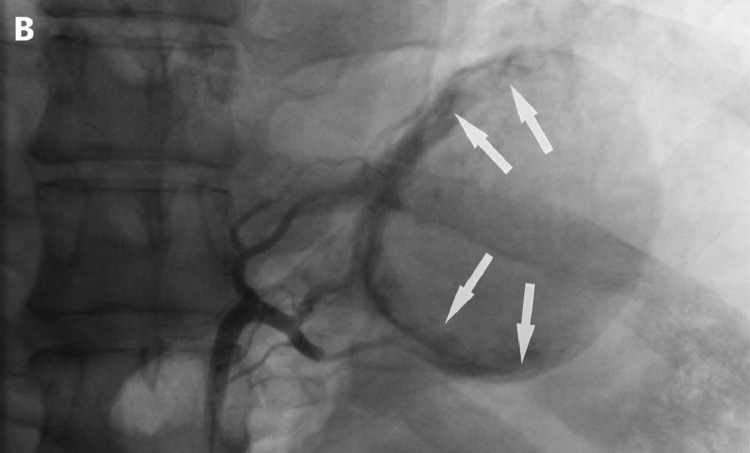
Left gastric angiogram Post-embolization, Stasis of flow: mild antegrade flow of contrast is seen due to collateral blood supply. (Black arrows)

**Fig. 3:**
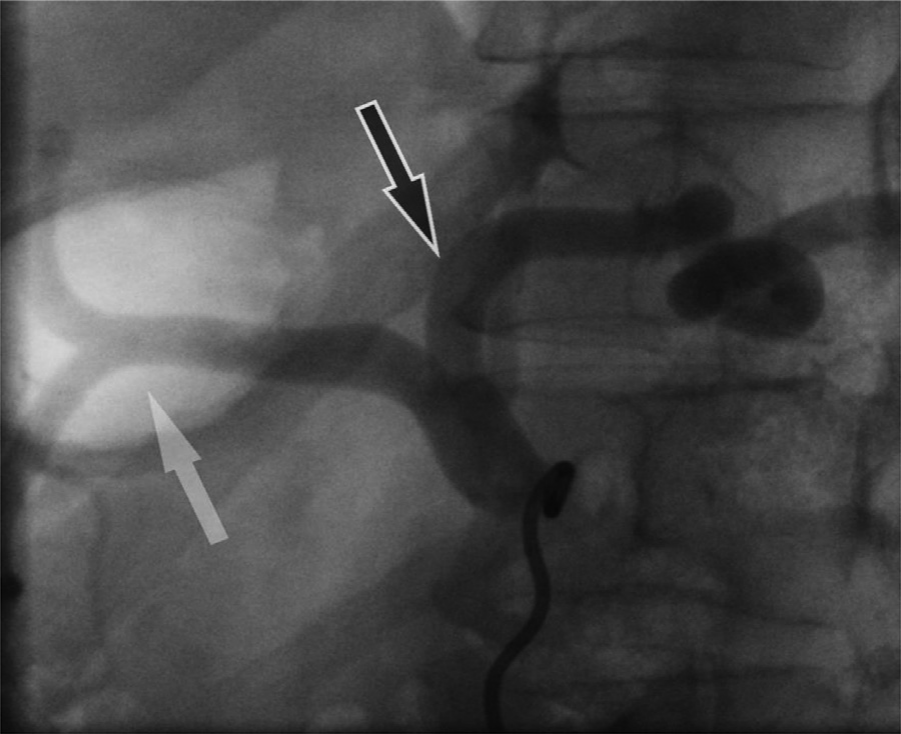
Left Gastric Artery (black arrow) shown clearly in this lateral off the celiac. Note the posterio-oblique angle makes selection challenging. The Celiac goes on to bifurcate into the Common hepatic and splenic arteries (white arrow)

**Fig. 4:**
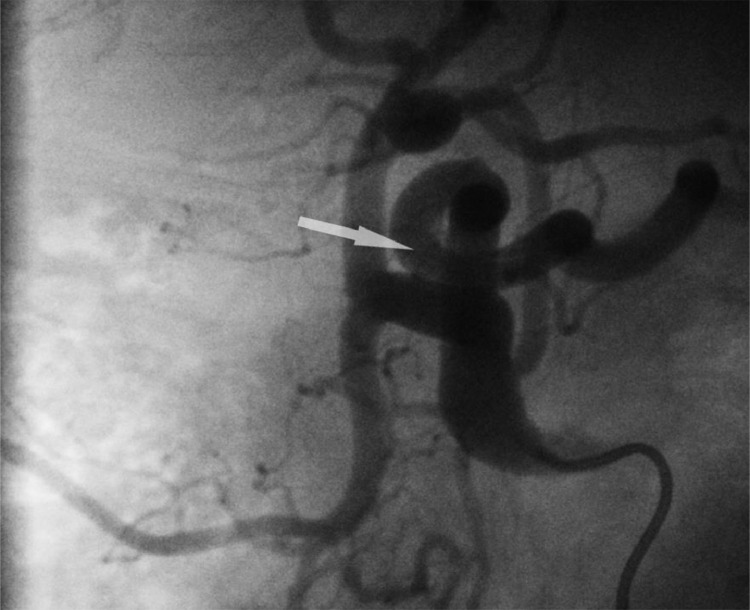
The Left Gastric artery (white arrow) and the false tripod is clearly visible in this angiogram proving a failed selection

**Fig. 5:**
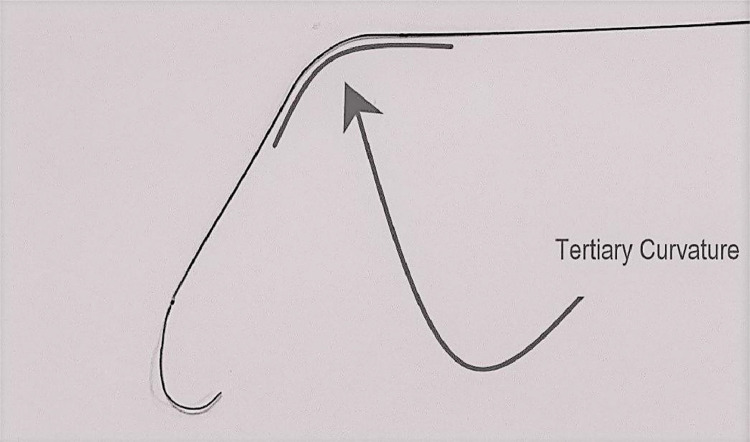
Actual Guide wire used, an ASAHI Prowater, steel-core guidewire. The hydrophobic spring coiled-tip preserves trackability, it is used often in coronary intervention

## Discussion

Typically, the left gastric artery arises directly superior from the celiac artery alongside the common hepatic and splenic artery at its termination - into what is known as a tripod and/or trifurcation. The anatomical variation in our case is known as a “false tripod” - defined as separate, often more proximal origin of anyone of these three branches. [6]

As the smallest branch of the trifurcation, the variety of anatomical variations and tendency to arise cephalad have all made the left gastric artery rather notorious for difficult catheterization. Even more so when there is a slightly posterior orientation. [7,8]

In the past Interventionalists have utilized loops (Waldman's loop), preformed catheters with and without coaxial wiring systems, and even catheter side-holes for arterial selection. Although in coronary literature adoption of secondary curvature is described, introducing a tertiary curve in a steel core guidewire for vascular access however has not [9]; we describe how a tertiary guidewire curve may be of aid in difficult catheterizations.
